# Effects of Oxygen Concentration and Culture Time on Porcine Nucleus Pulposus Cell Metabolism: An *in vitro* Study

**DOI:** 10.3389/fbioe.2019.00064

**Published:** 2019-04-02

**Authors:** Lukas M. Jaworski, Kelsey L. Kleinhans, Alicia R. Jackson

**Affiliations:** Orthopaedic Biomechanics Laboratory, Department of Biomedical Engineering, University of Miami, Coral Gables, FL, United States

**Keywords:** intervertebral disc, brachyury, gene expression, notochordal, consumption rate

## Abstract

Low back pain is a common ailment that affects millions of individuals each year and is linked to degeneration of the intervertebral discs in the spine. Intervertebral disc degeneration is known to result from an imbalance in anabolic and catabolic activity by disc cells. Due to the avascular nature of the intervertebral disc, oxygen deficiency may occur in the central nucleus pulposus (NP). The resulting hypoxia affects matrix regulation and energy metabolism of disc cells, although the mechanisms are not fully understood. This study investigates *in vitro* glucose consumption and gene expression by NP cells over time under varying oxygen tensions. Notochordal porcine NP cells were cultured in agarose discs at 21, 5, or 1% oxygen tension for 1, 5, or 10 days. The expression of 10 key matrix genes, as well as Brachyury (T), by NP cells was analyzed using RT-PCR. Glucose consumption was measured using a two-point method. Results show that culture time and oxygen tension significantly affect glucose consumption rates by porcine NP cells. There were also significant changes in T expression based on oxygen level and culture time. The 1% oxygen tension had a significantly higher T expression on day 10 than the other two groups, which may indicate a better maintenance of the notochordal phenotype. MMP 1 and 13 expression increased over time for all groups, while only the 5% group showed an increase over time for MMP 3. TIMP expression followed the direction of MMPs but to a lesser magnitude. Five percent and twenty-one percent oxygen tensions led to decreases in anabolic gene expression while 1% led to increases. Oxygen concentration and culture time significantly impacted glucose consumption rate and the gene expression of matrix regulatory genes with hypoxic conditions most accurately maintaining the proper NP phenotype. This information is valuable not only for understanding disc pathophysiology, but also for harnessing the potential of notochordal NP cells in therapeutic applications.

## Introduction

Lower back pain causes an enormous socioeconomic burden in many developed nations due to high direct and indirect costs (Deyo et al., 1991; Waddell, 1996; Maetzel and Li, 2002; Katz, 2006). Degenerative disc disease (DDD) of the intervertebral discs (IVD) of the spine is thought to be a major component in many cases of lower back pain, although the exact mechanisms of this degeneration are poorly understood (Urban and Roberts, 2003). Although degeneration is most commonly found in older individuals, affecting over 80% of those over 50, it has even been found in patients as young as teenagers (Miller et al., 1988; Adams and Roughley, 2006). Overall, disc degeneration is believed to be the result of improper matrix production by IVD cells, in which there is an imbalance in anabolic and catabolic activities (Adams and Roughley, 2006; Adams et al., 2009). Factors that influence the behavior of IVD cells include mechanical stimulation, osmotic pressure, nutrient levels, and pH, among others (Bibby et al., 2005; Grunhagen et al., 2006; Neidlinger-Wilke et al., 2006; Omlor et al., 2009; Rastogi et al., 2009; Guehring et al., 2010; Purmessur et al., 2013). With many potential contributing factors, elucidating which ones, or combinations thereof, initiate degeneration is an important task in order to better understand the pathways and mechanisms of DDD, and pose new treatment and/or regeneration modalities.

**Figure 1:**
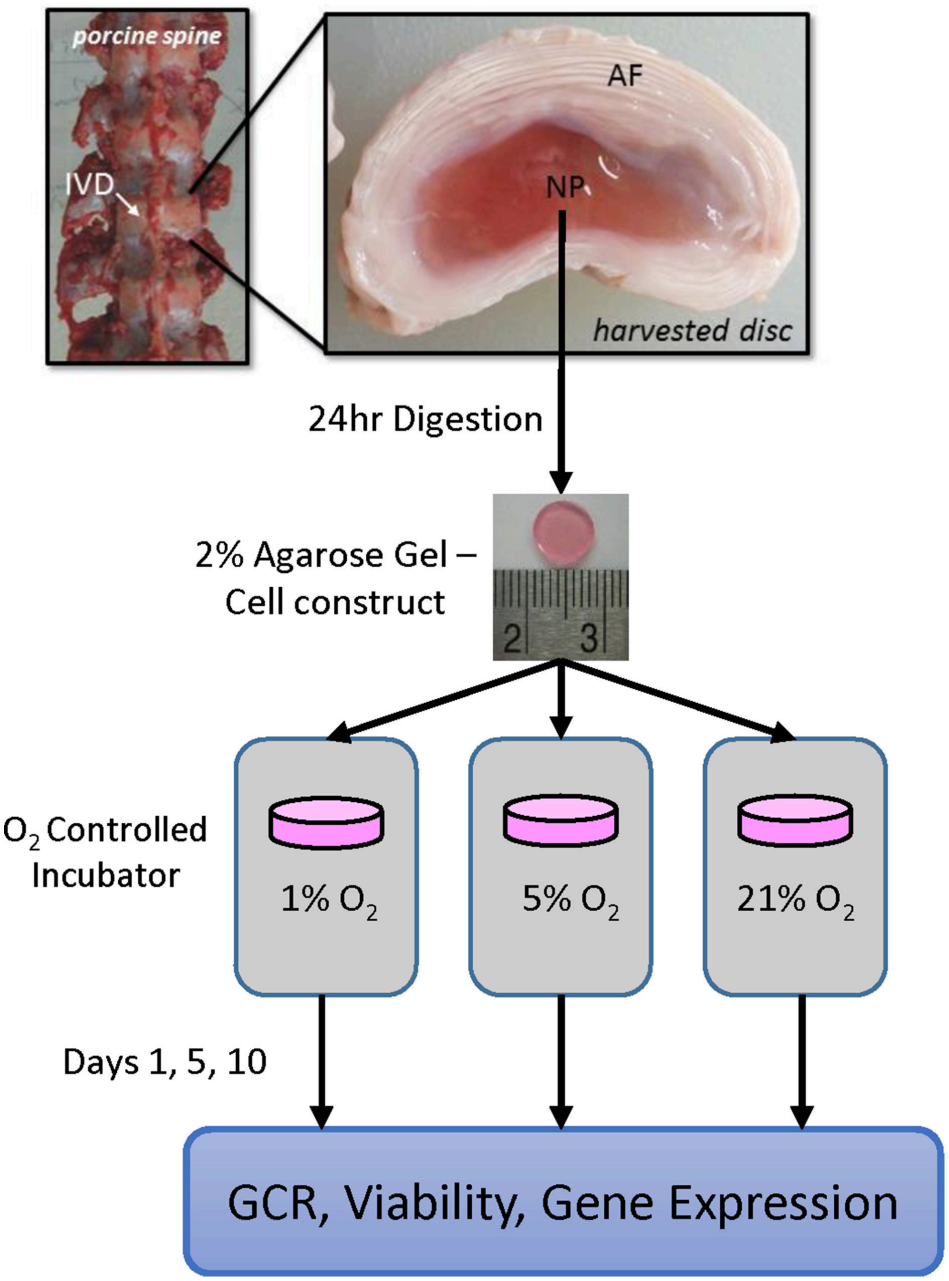
Schematic showing the experimental design, including harvest location and timeline. Annulus fibrous (AF) and nucleus pulposus (NP) regions are shown; cartilaginous endplate (CEP) is located at the interface between the disc and adjacent bony vertebrae (not shown).

The IVD is a complex tissue composed of the annulus fibrosis (AF) and the nucleus pulposus (NP), see Figure 1, as well as the cartilaginous endplates (CEPs) (not shown). The CEPs rest on the superior and inferior faces of the IVD and are the interface between the IVD and the vertebral body itself (Raj, 2008). Between the CEPs are the NP and AF regions, which work together to provide the major anatomic functions of the IVD: spinal flexibility and load distribution (Raj, 2008). While the cells of the CEP and the AF are derived from mesodermal origins, the NP of the IVD is heterogeneous with larger vacuolated cells originating from the notochord, termed notochordal NP cells, and chondrocytic NP cells whose origin is highly debated (Kim et al., 2003; Risbud et al., 2010a). The two predominant theories are that these chondrocytic cells are either phenotypically unique notochordal cells or cartilaginous cells that have migrated from the CEP (Kim et al., 2003; Cui et al., 2010; Risbud et al., 2010a). In humans the NP starts off with a high population of notochordal cells which gets replaced with chondrocytic cells within the first decade of life (Miller et al., 1988; Urban and Roberts, 2003; Adams and Roughley, 2006). This change in cell population is one of the major hallmarks of DDD, as notochordal cell disappearance coincides with the earliest signs of degeneration (Miller et al., 1988; Kim et al., 2003; Urban and Roberts, 2003; Adams and Roughley, 2006). Notochordal and chondrocytic NP cells produce matrix proteins in differing ratios, with notochordal NP cells assembling matrix proteins more similar to young NP tissue while chondrocytic NP cells produce matrix proteins closer to fibrocartilaginous cells (Cappello et al., 2006). Additionally, the notochordal NP cells were found to influence the behavior of their chondrocytic counterparts, altering their migration pattern, and matrix production (Erwin and Inman, 2006; Erwin et al., 2006; Kim et al., 2009). Notochordal cells within the NP are more metabolically intensive and proliferate at a slower rate than their chondrocytic counterparts (O'Halloran and Pandit, 2007; Kalson et al., 2008; Erwin et al., 2013; Kregar Velikonja et al., 2014). The porcine model was selected for this study due to its comparable size and geometry to human IVDs (Omlor et al., 2009). In addition the porcine NP contains notochordal cells in a high proportion, close to 80% for pigs of the same size and weight, well into adulthood and is useful for studying the behavior of notochordal cells in a large animal model (Hunter et al., 2003, 2004; Kim et al., 2003, 2009; Guehring et al., 2009; Omlor et al., 2009; Minogue et al., 2010; Gantenbein-Ritter and Chan, 2012).

The adult disc is avascular and relies on diffusion to move nutrients and metabolites through the extracellular matrix (ECM) (Adams and Roughley, 2006; Grunhagen et al., 2006). In lumbar IVDs the central NP is located several millimeters from the closest blood supply, creating steep nutrient gradients across the tissue (Crock et al., 1988; Adams and Roughley, 2006; Jackson et al., 2011a,b). Because of this, nutrient levels in the disc are often extremely low, with this problem being exacerbated by degenerative changes (Bibby and Urban, 2004; Bibby et al., 2005; Grunhagen et al., 2006; Adams et al., 2009; Jackson et al., 2011a,b). Varied nutrient and metabolite levels have been shown to have an effect on matrix turnover rates, metabolism, and gene expression in NP cells (Chen et al., 2002; Bibby and Urban, 2004; Bibby et al., 2005; Grunhagen et al., 2006; Neidlinger-Wilke et al., 2006; Adams et al., 2009; Rastogi et al., 2009). Establishing which genes are most affected by adverse nutrient conditions may elucidate targets for treatment of DDD and further increases the knowledge of the physiopathology of this debilitating condition.

The current treatment options for DDD are limited and for the most part highly conservative. Most focus on pain management and in some cases resort to invasive surgical treatments, such as spinal fusion or ablation of diseased tissue (Urban and Roberts, 2003; Adams and Roughley, 2006; Raj, 2008). These treatments do not currently address the root causes of the disease and in fact may accelerate the degeneration of neighboring discs (Urban and Roberts, 2003; Adams and Roughley, 2006; Raj, 2008). Understanding the behavior of NP cells can lead to expanded treatment strategies for the condition, including regenerative approaches to tackling this complex disease (O'Halloran and Pandit, 2007; Kalson et al., 2008; Erwin et al., 2013; Kregar Velikonja et al., 2014). In particular, notochordal NP cells are being heralded for their potential in disc regeneration efforts. In recent years, many studies have shown that co-culture of mature IVD cells or stem cells with notochordal cells can have a stimulating effect (Okuma et al., 2000; Yamamoto et al., 2004). Despite this, there still remains a lack of information about appropriate culture conditions for maintaining the notochordal cell phenotype. Understanding how key microenvironmental factors affect these cells is therefore useful in defining optimal culture conditions so that the regenerative capacity of notochordal cells can be fully harnessed.

One of the major components in matrix turnover is a class of proteins known as matrix metalloproteases (MMPs), which attack and degrade a diverse group of structural proteins (Nagase et al., 2006). These proteins are regulated by tissue inhibitors of metalloproteases (TIMPs), which bind to MMPs in a 1:1 ratio (Nagase et al., 2006). Dysregulation between these two classes of molecules has been found in both degenerated and herniated discs (Freemont et al., 2002; Weiler et al., 2002; Le Maitre et al., 2004; Kalichman and Hunter, 2008; Baillet et al., 2013). Low nutrient supply also negatively impacts anabolic processes and NP cells start to exhibit lower production or synthesize inappropriate matrix proteins (Urban and Roberts, 2003; Rinkler et al., 2010; Neidlinger-Wilke et al., 2012). Although this study uses healthy NP cells (i.e., not DDD), future studies monitoring these genes could lead to valuable insights into how notochordal NP cells respond to adverse conditions and what factors may initiate the degenerative cascade that leads to DDD. Limiting the production of MMPs would also facilitate ECM production, which would be highly beneficial in regenerative strategies, as most focus on replacing diseased tissue and cells with those that are more appropriate (Okuma et al., 2000; O'Halloran and Pandit, 2007).

This study aims to investigate the relationship between oxygen tension levels in the disc and the activity of notochordal NP cells. Specifically, we examined the glucose consumption rates, changes in expression of catabolic genes and their inhibitors, and the expression of important anabolic genes that occur over time. To do so, an agarose gel culture system was used to culture the notochordal NP cells at varied oxygen tensions (i.e., 1, 5, or 21%) over a culture period of 1, 5, or 10 days, as outlined in Figure 1. This system allows *in vitro* monitoring of the cells, with an ample notochordal NP cell population from a large animal, over a longer time frame, in order to study the impact that culture conditions have on the disc cells over time. This study will allow for a better understanding of the long-term response of the NP cells to differential oxygen conditions, which is useful not only in better understanding the pathophysiology of the IVD, but also for defining appropriate culture conditions for notochordal cells used for regenerative therapy. Moreover, quantitative results for cellular metabolic rates can be employed in computational modeling of the disc, in order to improve model prediction of *in vivo* conditions.

## Methods and Materials

### Cell Harvesting and Agarose Gel Seeding

Cells were harvested and cast into gels using previously reported protocols (Huang et al., 2007; Gonzales et al., 2014). Briefly, Yorkshire pigs 4–5 months of age (90–115 kg) were obtained from a local slaughterhouse (Cabrera Farms, Hialeah, FL). The spine was isolated within 2 h of death and the NP tissue was placed into an enzymatic solution, composed of high glucose (25 mmol/L) Dulbecco's Modified Eagle Medium (HG DMEM; Invitrogen Corp., Carlsbad, CA) supplemented with 0.6 mg/mL collagenase (Worthington Biochemical Corp., Lakewood, NJ) and 0.6 mg/mL protease (Sigma Chemical, St. Louis, MO), in order to digest the tissue for 24 h under continuous agitation. After enzymatic digestion, the solution was filtered through a 70-μm cell strainer (BD Biosciences, Bedford, MA). The suspension was then diluted, centrifuged, and resuspended to a concentration of 1 × 10^7^cells/mL in HG DMEM supplemented with 10% fetal bovine serum (FBS) (Atlanta Biologicals, Flowery Branch, GA), and 1% Antibiotic Antimycotic (AA) (Atlanta Biologicals). The suspension was then mixed with 4% agarose (Sigma Chemical), cast into custom molds (discs with *d* = 8 mm, *h* = 2 mm) with 100 μL per construct, and allowed to solidify, bringing the final constructs to 2% agarose gels containing 5 × 10^5^cells per gel. The cells were then cultured overnight in HG DMEM with 10% FBS and 1% AA at 37°C, 5% CO_2_. This 2% agarose gel model has been previously used to study the metabolism of porcine NP cells (Huang et al., 2007; Fernando et al., 2011; Salvatierra et al., 2011; Gonzales et al., 2014). Furthermore, agarose gels allow for unhindered diffusion of nutrients and the hydrogels also allow the cells to produce fully functional ECM (Gu et al., 2004; Smith et al., 2011).

### Glucose Consumption Rate Studies

Following the 24 h incubation at high glucose levels, the gels were divided into three groups depending on oxygen tension level. Groups were cultured in 5 mmol DMEM containing 10% FBS and 1% AA at 21% O_2_, 5% O_2_, or 1% O_2_ and 37°C, 5% CO_2_ in separate oxygen controlled incubators; this point marks Day 0. Media was changed every 2 days; new media was placed in the incubator an hour prior to media transfer to equilibrate the oxygen tension level. At 1, 5, and 10 days, gels were collected, cut into quarters, and placed into the individual wells of a 96-well plate. Two hundred microliter of 5 mmol glucose DMEM without FBS or AA was added and the gels were cultured for 4 h at 37°C and 5% CO_2_ at their respective oxygen tensions. The glucose consumption rate (GCR) of cells was determined by measuring the initial and final glucose concentrations using a custom modified glucose meter made from a commercially available blood glucose monitor and testing strips (Accu-Chek Aviva, Roche Diagnostics, Indianapolis, IN) and a sourcemeter (Model 2400, Keithley Instruments Inc., Cleveland, OH) to achieve high fidelity glucose measurements. Data were collected using a custom LabVIEW (National Instruments Corp., Austin, TX) script. Viability was assessed using Live/Dead^TM^ staining to ensure viability was kept above 90% (data not shown).

### Gene Expression Studies

Total RNA was extracted using trizol (Tri-Reagent, Molecular Research Center, Cincinnati, OH) protocol. Total RNA was quantified using the Qubit RNA BR assay kit (Life Technologies, Carlsbad, CA) and reverse transcribed to cDNA using the High Capacity cDNA reverse transcription kit (Applied Biosystems, Foster, CA), according to manufacturers' specifications. Gene expression was quantified through the use of RT-PCR (StepOnePlus, Applied Biosystems); a list of the genes studied can be found in Table 1. Multiple reference genes were used in order to reduce possible errors due to treatment conditions and random variations; a list of the reference genes used can be found in Table 2 (Vandesompele et al., 2002). Gene expression was compared using Δ*C*_q_ values. Unexpressed genes were assigned a C_q_ value of 40 to allow for statistical comparisons.

**Table 1:** List of studied genes^a^.

**Gene**	**Forward sequence**	**Reverse sequence**	**Amplicon length**	**Assentation number (GeneBank)**
TIMP 1	ATTTGTGGGAGCCCCAGAGT	CCCAAGGCATTGAACCCTTT	93	NM_213857.1
TIMP 2	CTACGGCAACCCCATCAAAC	AGGAGGGGGCCGTGTAGATA	101	NM_001145985.1
TIMP 3	CCTGCCTTGCTTTGTGACCT	CGGATGCAGGCGTAGTGTTT	102	XM_003126073.4
MT1 (MMP14)	CTGGACTGTCCGGAATGAGG	AGGGGTCATTGGAGTGCTCA	101	NM_214239.1
MMP 1	GGACCTGGAGGAAACCTTGCT	GCCTGGATGCCATCAATGTC	248	NM_001166229.1
MMP 3	TCCTGATGTTGGTTACTTCAGCAC	TTGACAATCCTGTAAGTGAGGTCATT	78	NM_001166308.1
MMP 13	CATGAGTTTGGCCATTCCTT	GTGGCTTTTGCCAGTGTAGG	90	XM_003129808.3
Aggrecan	CAGGTGAAGACTTTGTGGACATC	GTGAGTAGCGGGAGGAGCCC	450	NM_001164652.1
Collagen II A1	CACGGATGGTCCCAAAGG	TCGGGGCCTTTCTCACCAAC	150	XM_001925959.5
Brachyury	AAGTACGTGAACGGGGAGTG	CACGATGTGGATTCGAGGCT	213	XM_001928144.4

a *Forward and reverse primers, amplicon length, and ascension numbers for the genes used within the study*.

**Table 2:** List of reference genes^a^.

**Gene**	**Forward sequence**	**Reverse sequence**	**Amplicon length**	**Assentation number (GeneBank)**
18s	CGGCTACCACATCCAAGGA	AGCTGGAATTACCGCGGCT	188	NR_046261.1
GAPDH	GTTTGTGATGGGCGTGAACC	AGCTTGACGAAGTGGTCGTT	540	NM_001206359.1
ACTB	GTTCGAGACCTTCAACACGC	GCCTAGAAGCATTTGCGGTG	762	DQ845171
RPL4	ATACAGACCTTAGCAGAATCTTGA	AATCTTCTTGCGTGGTGCG	71	DQ845176
TBP	ACTGTGCTGCTATTTGGGCA	TGAAAACGCGGAATGTGTCTG	266	DQ845178
HPRT1	TATGGACAGGACTGAACGGC	TCCAGCAGGTCAGCAAAGAA	114	DQ845175
HMBS	CGAGAGTGCCCCTATGATGC	GTGTGTTGAGGTTTCCCCGA	172	DQ845174
YWHAZ	AAACAGCAGATGGCTCGAGAA	CTGCTTGTGAAGCATTGGGG	115	DQ845179

a *Forward and reverse primers, amplicon length, and ascension numbers for the reference genes used within the study*.

### Statistical Analysis

A two-way ANOVA was performed on the glucose consumption rate measurements with the independent variables being oxygen tension level and culture time. For significant results, a Tukey *post-hoc* test was used to determine significant differences between groups. Gene expression data were normalized against the panel of housekeeping genes using the NormqPCR package (v1.20.0) in R from Bioconductor (Perkins et al., 2012). ΔCq values were compared using a two-way ANOVA and Tukey for *post-hoc* analysis. All statistical analyses were carried out using R (v3.4.2) (Team, 2011).

## Results

**Figure 2:**
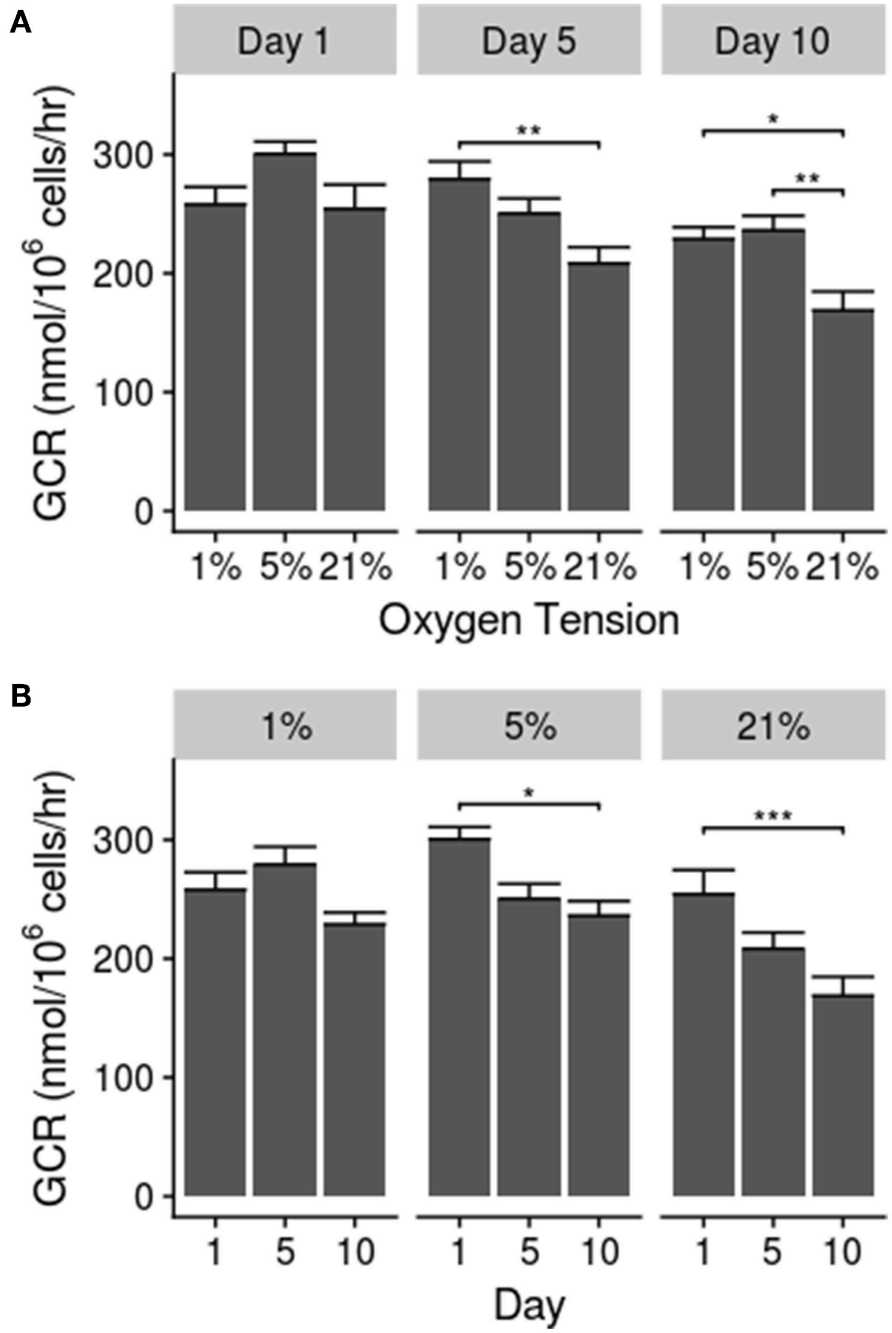
**(A)** Glucose consumption rate between groups over time. **(B)** Glucose consumption rate over time in each oxygen tension. Data presented as mean ± standard error of the mean; statistical significance shown as ^*^p ≤ 0.05, ^**^p ≤ 0.01, and ^***^p ≤ 0.001.

The results for glucose consumption rates are shown in Figure 2. The GCR showed a significant decline between days 1 and 10 for the 5% (*p* = 0.024) and 21% (*p* = 0.0004) oxygen tension groups. Additionally, the GCR for the 21% oxygen tension group was significantly lower than the 1% group on days 5 (*p* = 0.0058) and 10 (*p* = 0.043), and significantly less than the 5% group on day 10 (*p* = 0.0006). There was also a significant interaction between culture time and oxygen tension level (*p* = 0.0037) Significant differences between individual groups, as determined by *post-hoc* analysis, are noted on Figure 2.

**Figure 3:**
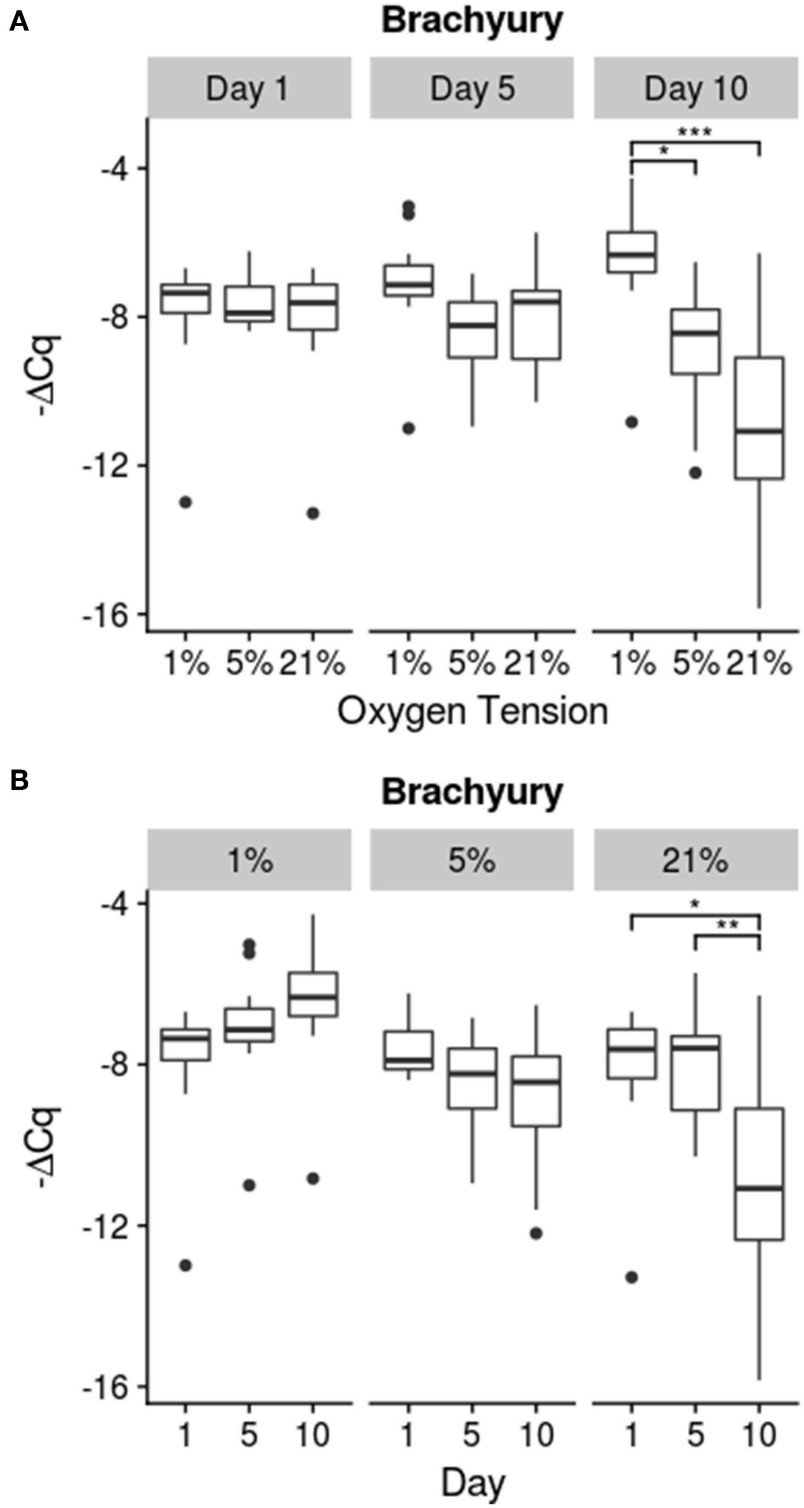
Brachyury (T) Expression. Relative expression is determined using the -ΔCq method. **(A)** compares oxygen tension within days while **(B)** shows changes over time within each oxygen tension; significance shown as ^*^p ≤ 0.05, ^**^p ≤ 0.01, and ^***^p ≤ 0.001.

Results for the expression of notochordal marker T are shown in Figure 3. Only the 21% oxygen tension group showed a significant decline in brachyury (T) expression on day 10, compared to day 1 (*p* = 0.012) and day 5 (*p* = 0.0071). The 5% group trended down over time while the 1% oxygen tension group trended up, but these trends were not found to be statistically significant. Additionally the 1% oxygen tension group had a significantly higher T expression than both the 21% (*p* ≪ 0.001) and 5% (*p* = 0.0032) groups on day 10. There was however a significant interaction between time and oxygen tension (*p* = 0.0008).

**Figure 4:**
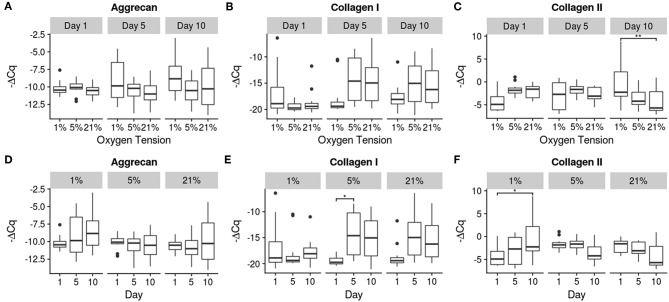
Gene expression of the anabolic genes using -ΔC_q_ values. The top row **(A–C)** contains between oxygen tension group comparisons over the days, while the bottom row **(D–F)** contains changes over time within each oxygen tension group. Significance shown as ^*^p ≤ 0.05 and ^**^p ≤ 0.01.

**Figure 5:**
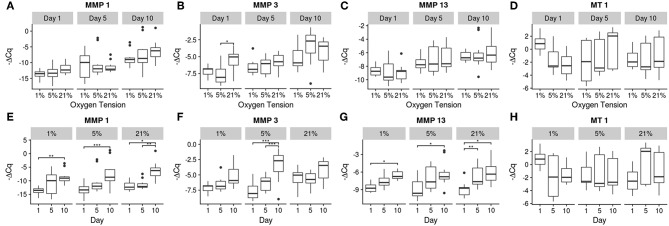
Gene expression of the catabolic genes using -ΔCq values. The top row **(A–D)** contains between oxygen tension group comparisons over the days, while the bottom row **(E–H)** contains changes over time within each oxygen tension group. Significance shown as ^*^p ≤ 0.05, ^**^p ≤ 0.01, and ^***^p ≤ 0.001.

**Figure 6:**
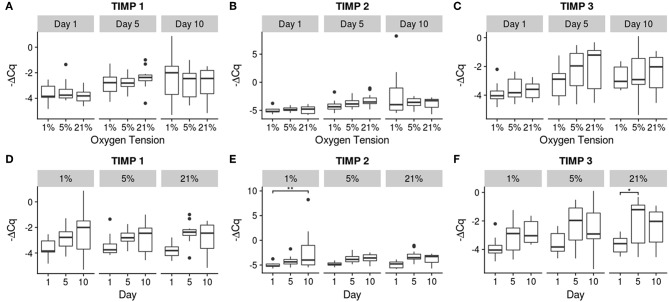
Gene expression of the catabolic inhibitor genes using -ΔCq values. The top row **(A–C)** contains between oxygen tension group comparisons over the days, while the bottom row **(D–F)** contains changes over time within each oxygen tension group. Significance shown as ^*^p ≤ 0.05 and ^**^p ≤ 0.01.

Relative gene expression for the 10 genes investigated is shown in Figures 4–6. There were no statistically significant differences noted in aggrecan expression, neither between oxygen tension levels nor over time. Collagen I expression showed a significant increase in expression between days 1 and 5 (*p* = 0.032) for the 5% oxygen tension group, with no other comparisons being significant, though the interaction term was significant (*p* = 0.045). Additionally collagen I was the only gene which had to have their C_q_ numbers set to 40 (i.e., undetectable expression). This was the case for the majority of samples, especially those analyzed on day 1, which showed no expression during RT-PCR. The 1% oxygen tension group showed a significant increase in collagen II expression between days 1 and 10 (*p* = 0.010) and had significantly higher expression of collagen II as compared to the 21% oxygen tension group on day 10 (*p* = 0.0089). The interaction term was also found to be significant (*p* = 0.0004).

MT 1 was the only catabolic gene which showed no significant differences in expression over time or between oxygen tension groups, but did show a significant interaction (*p* = 0.0096). The other catabolic genes had upward trends over time for all oxygen tension groups. Significant increases were found between days 1 and 10 for all oxygen tension groups for MMP 1 (*p* = 0.0073, 0.0004, and 0.0013 for 1, 5, and 21%, respectively) and additionally between days 5 and 10 for the 21% oxygen tension group (*p* = 0.0078). MMP 3 expression was higher on day 10 compared to days 1 (*p* ≪ 0.001) and 5 (*p* = 0.0006) for the 5% oxygen tension group. On day 1, the 21% oxygen tension group had significantly higher expression than the 5% oxygen tension group (*p* = 0.024). Additionally the interaction term was found to be significant (*p* = 0.0232). For MMP 13, all oxygen tension groups showed increases in expression between days 1 and 10 (*p* = 0.047, 0.0049, and 0.0016 for 1, 5, and 21%, respectively), with the 21% oxygen tension group showing an increase between days 1 and 5 as well (*p* = 0.049).

Though the TIMPs showed similar trends compared to the MMPs, the increases were milder with only TIMP 2 showing a significant increase in the 1% oxygen tension group between days 1 and 10 (*p* = 0.0034) and TIMP 3 in the 21% oxygen tension group between days 1 and 5 (*p* = 0.040). Significant differences between individual groups and reference levels are noted in the figures.

## Discussion

The overall goal of this study was to investigate the effects of oxygen tension levels and culture time on the activity and metabolism of notochordal NP cells. To our knowledge, there are few *in vitro* experiments tracking the changes in metabolism of NP cells during prolonged culture. In order to examine the effect of culture time on cell activity, we investigated the response of NP cells to different oxygen tension levels for up to 10 days in culture. NP cells need long culture times in order to produce cells in therapeutic quantities. Thus, understanding how to better maintain the NP cell phenotype over longer culture timeframes will become a valuable insight. Although several studies have been done over long periods *in vivo*, these studies have been mostly carried out in rabbits and other small animals, with a few large animal studies in the literature (Olmarker et al., 1993; Sobajima et al., 2005; Hoogendoorn et al., 2008; Guehring et al., 2010). Results from small animal studies are difficult to interpret given that low nutrient levels may not be typically present in small discs. The porcine model may be useful in tracking the early signs of degeneration as pigs maintain a majority of their notochordal cells well into adulthood (Hunter et al., 2003, 2004; Guehring et al., 2009; Omlor et al., 2009; Gantenbein-Ritter and Chan, 2012). Changes in the cell population and productivity have been found in models of simulated degeneration (Maldonado and Oegema, 1992; Omlor et al., 2009; Purmessur et al., 2013). Degenerate discs display a higher proportion of chondrocytic cells compared to notochordal cells; the origin of these chondrocytic cells is not yet firmly established nor the mechanism(s) which cause the notochordal cells to disappear (Waddell, 1996; Hoogendoorn et al., 2008; Guehring et al., 2009, 2010; Omlor et al., 2009; Risbud et al., 2010a; Purmessur et al., 2013).

To track the lineage of the cells of the IVD, notochord specific markers, such as sonic hedgehog, cytokeratin 8, or brachyury (T), can be used (Minogue et al., 2010; Risbud et al., 2010a). These genes have been found to be highly specific to the notochordal cell lineage and are maintained throughout the lifetime of the cell (Minogue et al., 2010; Risbud et al., 2010a). For these experiments, the T gene was used to monitor lineage stability of the notochordal NP cells. As the transcription factor T is critical for the maintenance of the notochordal cell lineage, a decrease in expression may be a signal that the notochordal cells are beginning to dedifferentiate. The general morphology of the harvested NP cells indicated a high proportion of cells which fit the notochordal morphology and good cell viability, although an exact quantification was not carried out. Also porcine NPs usually contain a majority of notochordal cells even into maturity (Hunter et al., 2003, 2004; Guehring et al., 2009; Omlor et al., 2009; Gantenbein-Ritter and Chan, 2012). The native stem cell of the NP niche does not appear to be of notochordal origin and may be another source of the chondrocytic cells (Erwin et al., 2013). Though the origin of the chondrocytic cells is still debated, monitoring the T transcription factor should give an indication as to the stability of the notochordal cell population within the disc. As seen in Figure 3, the 1% oxygen tension group had the highest expression of T compared to the 21 and 5% oxygen tension groups. Moreover, the expression of T for the 21% oxygen tension group showed a significant decrease in expression at day 10. This could indicate that lower oxygen tensions are better at preserving the notochordal phenotype while atmospheric conditions are inappropriate for culturing and expanding NP cells. As oxygen tension is an important consideration in differentiating stem cells to a chondrocytic lineage, it may also be an important factor in maintaining the phenotype of the NP cell (Risbud et al., 2010b; Gorth et al., 2015; Naqvi and Buckley, 2015). Oxygen tension has also been shown to differentially stimulate protein production within bovine caudal discs (Ishihara and Urban, 1999). Previous studies have found that the metabolism of NP cells is mostly anaerobic with the cells exhibiting low sensitivity to oxygen deprivation while low glucose can cause rapid declines in viability (Horner and Urban, 2001; Bibby and Urban, 2004; Bibby et al., 2005; Grunhagen et al., 2006; Huang et al., 2007; Stephan et al., 2011).

There are differences in metabolism between notochordal and chondrocytic NP cells (Maldonado and Oegema, 1992; Huang et al., 2007; Guehring et al., 2009). The notochordal NP cells produce large quantities of proteoglycans that impart the ECM of NP with high water content and swelling pressure, which is vital to disc function; by comparison, chondrocytic NP cells produce a more collagenous, and less hydrated ECM (Walmsley, 1953; Trout et al., 1982; Hunter et al., 2004). Moreover, notochordal NP cells are able to regulate ECM production of the chondrocytic NP cells (Cappello et al., 2006; Adams et al., 2009; Gantenbein-Ritter and Chan, 2012; Naqvi and Buckley, 2015). These properties of notochordal cells make them an attractive target for regenerative strategies, despite their low proliferation rate (O'Halloran and Pandit, 2007; Kalson et al., 2008; Erwin et al., 2013; Kregar Velikonja et al., 2014). Several studies have used co-culture systems to increase the proliferation rate of NP cells, which mitigates some of this drawback (Okuma et al., 2000; Yamamoto et al., 2004). Also, changes in the nutrient microenvironment can lead to aberrant expression in matrix and matrix degrading proteins (Ishihara and Urban, 1999; Freemont et al., 2002; Weiler et al., 2002; Le Maitre et al., 2004; Bibby et al., 2005; Sobajima et al., 2005; Neidlinger-Wilke et al., 2006, 2012; Rastogi et al., 2009). Therefore, while oxygen may not be a vital nutrient in maintaining cell viability, it does play a role in maintaining cell phenotype. This is evident by the 1% oxygen tension group having the lowest expression of matrix degrading enzymes and being the only group to upregulate matrix molecule production. Furthermore, this group was the only one which did not see collagen type I becoming more expressed over time.

The GCRs found in this study fall in the range of those reported in the literature (Holm et al., 1981; Ishihara and Urban, 1999; Bibby et al., 2005; Guehring et al., 2009; Salvatierra et al., 2011), with values in the range from ~100 to 300 nmol/10^6^ cells/h for porcine and bovine NP cells, which vary based on treatment conditions, such as mechanical stimulation and pH (Bibby et al., 2005; Guehring et al., 2009; Salvatierra et al., 2011). Furthermore, the interaction effect found in the two-way ANOVA indicates that the NP adapts to the new oxygen tension over time. Other studies utilizing bovine and adult human cell sources report lower GCRs than those found here, but the cell population of these cell sources is predominantly chondrocytic and does not contain the high proportion of notochordal cells that porcine sources do (Hunter et al., 2003, 2004; Bibby et al., 2005; Omlor et al., 2009; Gantenbein-Ritter and Chan, 2012; Cisewski et al., 2017). Numerous investigators have developed computational models that incorporate cellular activity, including metabolic rates (Malandrino et al., 2015). The values for the GCR found here can be used to further refine such computer models of the IVD to better simulate the conditions within the disc itself.

Previous studies investigating the effects of oxygen tension on glucose metabolism by IVD cells have had mixed findings, showing both positive and negative Pasteur effects; these studies include both notochordal and chondrocytic NP cell populations (Holm et al., 1981; Ishihara and Urban, 1999; Bibby et al., 2005; Guehring et al., 2009). At higher oxygen tensions there seemed to be an adaptation in the glucose consumption rate over time, possibly becoming more aerobic with higher oxygen exposure. These findings are similar to earlier findings reported in the literature (Ishihara and Urban, 1999). A previous study by Guehring et al. (2009) found that lactate production rates by notochordal cells were ~22–27% higher in 1% oxygen tension as compared to 21% oxygen tension; lactate production generally corresponds to glucose consumption in a ~2:1 ratio, with the range in the literature going from 1.7 to 2.2 (Holm et al., 1981; Ishihara and Urban, 1999; Razaq et al., 2003; Bibby and Urban, 2004; Bibby et al., 2005; Guehring et al., 2009; Cisewski et al., 2017). In addition to the 21% oxygen tension group, the 5% oxygen tension group experienced a significant, although less marked, decline in the GCR over time. The trend seen in this study could potentially be a progressive adaptation over time to a more aerobic environment, even though glycolysis is still the most important method of energy extraction within the NP (Holm et al., 1981; Ishihara and Urban, 1999; Bibby et al., 2005). The 1% oxygen tension group, on the other hand, showed no particular trend over time which may be due to the disc environment normally being anaerobic (Huang and Gu, 2008).

The overall trend of gene expression tended toward catabolism, with many more catabolic genes increasing expression compared to anabolic expression, which could indicate degenerative changes in the cells. A study by Weiler et al. (2002) found that concentrations of MMPs 1, 2, 3, and 9 tended to be higher around clefts and tears in the IVD of surgical patients and cadaveric donors (Weiler et al., 2002). Since the MMPs were clustered around areas where degeneration actually occurred, there is some indication of a correlation between the two, though causality is difficult to determine from this earlier study. The expression of MMP 1 increased in all groups, with its primary function being the breakdown of collagens I, II, III (Tallant et al., 2010). This may indicate a remodeling of the collagen network as there was also a corresponding increase in collagen I expression in both the 5 and 21% oxygen tension groups. The 1% oxygen tension group did upregulate collagen II expression between days 1 and 10, and produced significantly more collagen II than the 21% oxygen tension group, which is what the healthy NP would normally contain (Erwin and Hood, 2014). Aggrecan expression was unaffected by oxygen tension; this may be due to a regulatory mechanism which is resistant to changes in oxygen tension (Agrawal et al., 2007). MMP 13 was also upregulated over time for all groups; this MMP preferentially breaks down collagen II and again indicates a matrix remodeling response of the NP cells (Leeman et al., 2002). MMP 3 expression was only significantly increased within the 5% oxygen tension group, though all of the oxygen tension groups trended upward. MMP 3 also targets collagen II preferentially, and is the most abundant MMP natively expressed within the disc (Cui et al., 2010; Tallant et al., 2010; Baillet et al., 2013).

Gene therapy with BMP 2 or TIMP 1 has been shown to slow the progression of degeneration (Wallach et al., 2003; Leckie et al., 2012), indicating that the NP has a means to protect itself from the spiral of degeneration via inhibition of catabolism through the use of TIMPs. Although only the 1 and 21% oxygen tension groups showed significant increases for TIMP 2 and 3, respectively, all of the TIMPs had upward trends over time. The trends seen in this study indicate that NP may be trying to regulate the effect of the MMPs by producing TIMPs to offset their effect. Though there is some co-upregulation of the TIMPs with MMPs, TIMPs are up-regulated to a smaller extent than the MMPs. Since the TIMPs are only able to do a 1:1 inhibition of the MMPs, the stronger upregulation of MMPs indicates a net gain of catabolic activity. This could, in turn, lead to an imbalance in matrix production and degradation, thus initiating degeneration in the IVD. This would also not be favorable for generating tissue constructs with the appropriate matrix for therapeutic applications.

It should be noted that although there were differences among the treatment conditions, as seen in Figures 4–6, the effect of time had a much greater influence on the relative expression of the various genes as compared to the effect of oxygen tension level. Most genes tended to show greater changes over time, with decreasing expression of matrix building transcripts, strong upregulation of MMPs, and insufficient inhibitory activity to offset these changes. Only the 1% oxygen tension group, which had smaller changes to the catabolic genes and the only significant anabolic expression increases over time, showed any sign of balancing catabolism and anabolism. The day 1 trend seemed to roughly mimic other short term studies done under low oxygen conditions (Neidlinger-Wilke et al., 2012). A long term study of bovine NP cells in agarose discs showed similar trends in collagen type II expression trends over time (Gorth et al., 2015). This indicates that low oxygen tension is best able to maintain the NP cell phenotype, which was also observed in other studies in the literature, with hypoxic conditions being more favorable (Risbud et al., 2005; Mwale et al., 2011; Gorth et al., 2015). All oxygen tension groups showed increases in MMP 1 and 13 expressions with time. This could be due to the lack of key environmental factors, such as mechanical cues, which play an important role in balancing cellular metabolism (Adams and Roughley, 2006; Neidlinger-Wilke et al., 2006; Guehring et al., 2010; Purmessur et al., 2013).

There are several limitations to the present study which should be noted. First, only limited subsets of anabolic and catabolic genes were studied. Some of the other collagen subtypes, such as III, V, VI, IX, and XI, might play a role in degeneration even though they are not the major collagens expressed within the disc (Cassinelli et al., 2001). Additionally, the ADAMs family of metalloproteases, which target and degrade many matrix proteins, were not covered in this study (Seals and Courtneidge, 2003). Glycosaminoglycan content was also not directly measured, so although the gene expression indicated catabolism, there is no direct verification of this process happening. Additionally, the direct verification of notochordal cells via staining was not carried out and would be useful to incorporate in future studies. Nonetheless, we believe that the genes investigated here are representative of the overall trend of activity of NP cells under the conditions investigated. Furthermore, no previous study has investigated the effects of culture conditions on such a large number of genes.

In the future, we will incorporate mechanical loading schemes in order to study the response under varied load and nutrient conditions, as this would be more indicative of the *in vivo* environment. Also, future studies should employ whole disc explants to better preserve and mimic *in vivo* conditions while still providing the level of control of an *in vitro* experiment. These explants would also allow for histological staining and quantifying glycosaminoglycan content in order to better monitor degenerative progress over time.

In conclusion, this study showed that, in agarose disc culture, oxygen tension levels can play a vital role in maintaining proper expression levels of matrix regulation genes of notochordal NP cells. In order to limit degradation, the cells have a native protection scheme in place, the TIMPs, which may become overwhelmed by the large overabundance of MMPs. Hypoxic conditions were best suited for maintaining cell phenotype and limiting aberrant matrix degradation. The notochordal marker T showed significant differences in its expression by day 10, indicating that hypoxic conditions may be better suited for maintaining notochordal cell phenotype within the NP. Glucose consumption, on the other hand, did change with time in culture and also had significant differences in GCR between oxygen tension groups. Overall, this study provides valuable insight into the role of the nutritional environment in maintaining healthy NP cell expression, including a detailed look into the behavior (e.g., metabolism, gene expression) of NP cells, with a high proportion of notochordal cells, over time with cells from a large animal. The general trend of change within the disc seen here, coupled with future studies characterizing protein or ECM composition, will allow for a more detailed look at how the NP and IVD respond to adverse conditions, and allow us to provide a more optimized environment for culturing these cells. Quantitative information on GCR gained from this study can also be used to improve theoretical prediction of the *in vivo* nutritional environment in the disc, which can be harnessed to develop treatment strategies for disc regeneration.

## Data Availability

The datasets generated for this study are available on request to the corresponding author.

## Author Contributions

All authors were involved in the conception and design of this study. LJ collected all data, performed statistical analysis, and wrote the initial draft of the manuscript. AJ assisted in the preparation and critical review of the manuscript. All authors approved the final version of the manuscript and take responsibility for the findings of this study.

### Conflict of Interest Statement

The authors declare that the research was conducted in the absence of any commercial or financial relationships that could be construed as a potential conflict of interest.
